# Individualised progesterone receptor modulator prevention strategies for triple-negative breast cancer in *BRCA1* pathogenic variant carriers

**DOI:** 10.1038/s41523-026-00998-x

**Published:** 2026-06-25

**Authors:** Martin Widschwendter, Rita Schmutzler, Nora Pashayan, Jelle Wesseling, Marc Tischkowitz, Michal Zikan, Miranda Steenbeek, Marike Gabrielson, Christian F. Singer, Cathrin Brisken, Rebecca Gomperts, Sharon Cameron, Dafydd Gareth Evans, Andri Papakonstantinou, Marc Buyse, Kerstin Rhiem, Mateja Krajc, Charlotte Vavourakis, Elisa Redl, Chiara Herzog, James Barrett, Mohammed Fatih Rasul, Deborah Utjés, Kiriaki Papaikonomou, Angelique Flöter Rådestad, Athanasios Zouzos, Twana Alkasalias, Kristina Gemzell-Danielsson

**Affiliations:** 1 The Daffodil Centre, Cancer Council New South Wales and University of Sydney, Sydney, Australia; 2 European Translational Oncology Prevention and Screening Institute, Universität Innsbruck, Innsbruck, Austria; 3 Institute for Biomedical Ageing Research, Universität Innsbruck, Innsbruck, Austria; 4 Tirol Kliniken, Hall in Tirol, Austria; 5 Department for Women’s Health, University College London, London, UK; 6 Department for Women’s and Children’s Health, Karolinska Institutet, Stockholm, Sweden; 7 Centre for Hereditary Breast and Ovarian Cancer and Centre for Integrated Oncology (CIO), Faculty and University Hospital Cologne, University of Cologne, Cologne, Germany; 8 Centre for Cancer Genetic Epidemiology, Department of Public Health and Primary Care, University of Cambridge, Cambridge, UK; 9 Division of Molecular Pathology and Department of Pathology, Netherlands Cancer Institute, Amsterdam & Department of Pathology, Leiden University Medical Center, Leiden, The Netherlands; 10 Department of Genomic Medicine, National Institute for Health Research Cambridge Biomedical Research Centre, University of Cambridge, Cambridge, UK; 11 Department of Gynecology and Obstetrics, Bulovka University Hospital and First Faculty of Medicine, Charles University, Prague, Czech Republic; 12 Department of Obstetrics and Gynecology, Radboud University Medical Centre, Nijmegen, The Netherlands; 13 Department of Medical Epidemiology and Biostatistics, Karolinska Institutet, Stockholm, Sweden; 14 Department of Obstetrics and Gynaecology and Comprehensive Cancer Center, Medical University of Vienna, Vienna, Austria; 15 ISREC - Swiss Institute for Experimental Cancer Research, School of Life Sciences, Ecole Polytechnique Federale de Lausanne (EPFL), Lausanne, Switzerland; 16 Division of Breast Cancer Research, The Breast Cancer Now Toby Robins Research Centre, The Institute of Cancer Research, London, UK; 17 Women on Waves, Amsterdam, Netherlands; 18 Chalmers Centre for Sexual and Reproductive Health, NHS Lothian, Edinburgh, UK; 19 Centre for Reproductive Health, Institute for Regeneration and Repair, University of Edinburgh, Edinburgh, UK; 20 University of Manchester, School of Biological Sciences, Division of Evolution, Infection and Genomics, Manchester, Greater, Manchester, UK; 21 Department of Oncology-Pathology, Karolinska Institutet, Stockholm, Sweden; 22 Department of Breast Cancer, Endocrine Tumours, and Sarcoma, Karolinska Comprehensive Cancer Center, Stockholm, Sweden; 23 IDDI (International Drug Development Institute), Louvain-la-Neuve, Belgium; I-BioStat, Hasselt University, Hasselt, Belgium; 24 Cancer Genetics Clinic, Institute of Oncology Ljubljana, Zaloška 2, 1000 Ljubljana, Slovenia; University of Ljubljana, Kongresni trg, Ljubljana, Slovenia; 25 Department of Pharmaceutical Basic Science, Faculty of Pharmacy, Tishk International University, Erbil, Iraq; 26 Department of Hereditary Cancer, Theme Cancer, Karolinska Comprehensive Cancer Center, Stockholm, Sweden; 27 Department of Oncology and Pathology, Karolinska Institutet, Stockholm, Sweden; 28 General Directorate of Scientific Research Center, Salahaddin University-Erbil, Erbil, Iraq; 29 WHO Collaborating Centre, Division of Gynecology and Reproduction, Karolinska University Hospital, Stockholm, Sweden

**Keywords:** Cancer, Genetics, Oncology

## Abstract

Women with germline *BRCA1* pathogenic variants face a very high risk of early-onset triple-negative breast cancer (TNBC), with risk-reducing mastectomy currently the only established preventive option. Emerging evidence suggests aberrant progesterone signalling contributes to TNBC risk, while preclinical studies indicate progesterone receptor modulators such as mifepristone may substantially reduce cancer development. DNA methylation-based markers reflecting field cancerization and replicative age could enable real-time monitoring of preventive efficacy and support an individualised primary prevention approach.

## Introduction

Breast cancer (BC) is the leading cause of cancer-related death among young women worldwide, and its prevalence is increasing^1^. Pathogenic variants (PVs) in the *BRCA1* gene are responsible for approximately 1 to 3% of BCs globally, with 60 to 70% of these cancers being of basal cell origin and lacking expression of estrogen, progesterone, and HER2 receptors, making them triple negative breast cancers (TNBC)^2^. Among all BC subtypes, TNBC has the worst prognosis^3^ and treatment options remain limited due to the absence of therapeutic targets. *BRCA1* PV carriers have a 40% lifetime risk for TNBC, compared to a 1% lifetime risk in women without PVs; notably, this risk rises sharply from 25–30 years onwards^2^. Additionally, *BRCA1* PV carriers have a 45% lifetime risk of ovarian cancer (OC), compared to 1.8% risk for women without PVs and the risk starts to rise from the age of 35 onwards^4^.

Options for management of TNBC risk for *BRCA1* PV carriers are rather scarce. Primary prevention strategies (aimed at preventing cancer development) are mostly limited to risk reducing bilateral mastectomy, which can lower BC risk by 90-95%^5^ and remains the gold standard for breast cancer prevention. However, the procedure shows only a tendency towards improved survival^6^, has a higher risk of failure in women who smoke or have a high body mass index (BMI), carries a risk of post-surgical complications, and has negative consequences on self-perception and physical and mental well-being. Risk reduction through selective estrogen receptor modulators (SERMs; e.g. tamoxifen and raloxifene) and aromatase inhibitors^7,8^ has shown efficacy in reducing estrogen receptor positive BC risk, but not mortality, and these drugs do not prevent TNBC.

Secondary prevention strategies (aimed at early cancer detection) are currently limited to enhanced imaging-based surveillance (i.e. mammograms and breast MRI) and these remain a core aspect of the follow-up management of *BRCA1* PV carriers. Blood-based early detection strategies (e.g. detection of circulating tumor DNA) have not shown great promise so far due to limited sensitivity^9,10^.

Individual BC risk for *BRCA1* PV carriers varies considerably, making risk stratification an important component of both primary and secondary prevention. These risks have been substantiated in recent years by comprehensive cohort studies. In particular, age-dependent prospective incidence rates have been empirically determined and allow age-dependent risk prediction^11,12^. This has significantly improved risk communication and informed decision-making on the uptake of preventive measures. CanRisk, an online tool based on personal, family and genetic risk factors, is now fully embedded in many cancer genetics services and implementation studies using CanRisk with other risk factors [i.e. polygenic risk score (PRS) and non-genetic risk factors] are underway in tertiary care (e.g. Precision HBOC, CanRisk-Clingen) and primary care (e.g. CanRisk-GP). Such comprehensive risk prediction has been shown to also modify individual breast cancer risk in *BRCA1* PV carriers that might provide additional information for decision-making regarding prophylactic bilateral mastectomy^13^. Although mammographic density is a risk factor for BC, it is currently not well integrated into risk stratification, and – in particular for *BRCA1* PV carriers – might be unsuitable due to lower mammographic density observed in TNBC^14^.

## The unmet needs

**Fig. 1:**
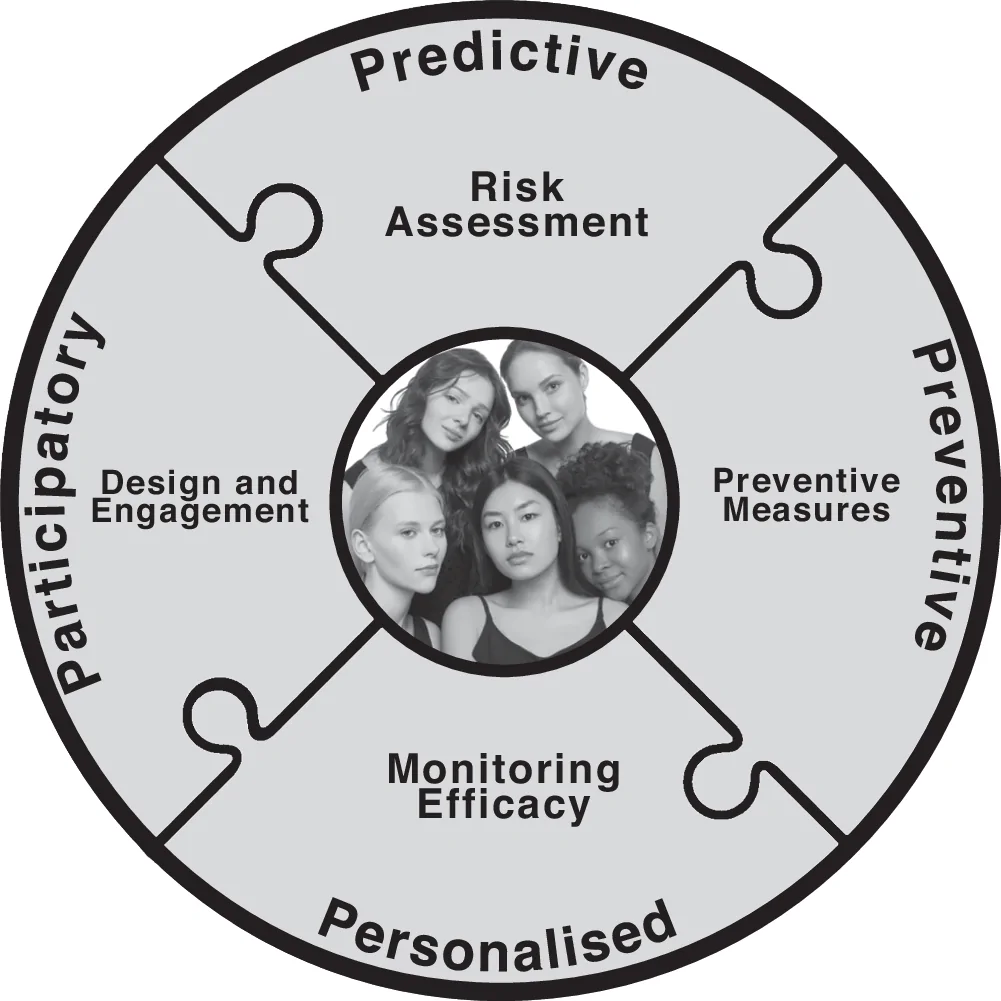
Principles of P4 Medicine applied to primary breast cancer prevention in *BRCA1* pathogenic variant carriers. The image in the centre of the Figure has been obtained from Adobe Stock.

The principles of P4 Medicine (Fig. 1) provide a structured approach to highlight the several unmet needs required to advance primary BC prevention in *BRCA1* PV carriers.

1. Prediction: Although the above-mentioned tools are available to refine BC risk, none of them indicate how this risk changes over time or can be influenced by interventions. *BRCA1* is ubiquitously expressed and centrally involved in DNA repair, however, until recently, only cell-autonomous mechanisms (i.e. direct effects of the *BRCA1* PV status in the cancer’s cell of origin such as reduced DNA repair in breast epithelial cells) were considered as pathogenic factors in *BRCA1* PV carriers^15^. This is at odds with the observation that carcinogenesis is limited to tissues without particularly high cell turnover (i.e. breast and Fallopian Tube fimbriae epithelial cells); organs with a high cell turnover (e.g. intestine) and thereby a high demand for accurate DNA repair are not at risk. Evidence has now shown that cell non-autonomous factors play a central role and that their local effects might serve as ideal risk predictors.
2. Prevention: Apart from surgical removal of the organs at risk, no primary preventive measures have been proven to reduce TNBC risk in *BRCA1* PV carriers. Targeting cell non-autonomous factors could offer a promising alternative.
3. Personalised: The lack of means by which to measure the effectiveness of a primary preventive intervention has resulted in therapeutic options being offered without personalisation regarding the modality, the dosage or duration. To gain sufficient coverage and willingness for primary prevention, personalised efficacy measurements are needed.
4. Participatory: Clear communication, decision aids^16^, decision coaching and simplicity of risk assessment, preventive measures and monitoring are essential to ensure high uptake, long term engagement, adherence and commitment from the *BRCA1* PV carrier.

## Progesterone – a key risk factor for TNBC

Elevated steroid hormone levels are one of the most striking recently discovered cell non-autonomous factors mediated by a pathogenic *BRCA1* PV. These findings point to elevated progesterone levels during the luteal phase of the menstrual cycle^17–20^. Beside several other effects, progesterone stimulates Receptor Activator of Nuclear factor Kappa-B Ligand (RANKL) secretion^21^ in hormone-sensitive mature luminal cells, which triggers the proliferation of luminal progenitor (and basal) cells via a paracrine mechanism^22–25^. These estrogen- and progesterone-receptor-negative cells are the cells of origin for TNBC.

Additional evidence supporting progesterone as the most important steroid driving the formation of BCs with poor prognosis includes findings from the Women’s Health Initiative study^26^ where the combination of estrogen with medroxyprogesterone acetate for hormone replacement was associated with an increased BC risk, while estrogen alone was linked to a reduced risk. In addition, long-term use of oral contraceptive pills, mainly due to the progestin component^27^, increased the TNBC risk^28^. Higher parity, which corresponds to prolonged exposure to progesterone, was associated with higher TNBC risk among black women^28^. Most recent evidence further supports progesterone’s key role in the formation of TNBC also in young women: Estrogen plus progestin hormone therapy was associated with an increased incidence of young-onset breast cancer, with a particularly strong association observed for TNBC (HR 1.50; 95% CI, 1.02–2.20) whereas estrogen alone decreased the breast cancer risk significantly (HR 0.86; 95% CI 0.75–0.98)^29^.

The observation that risk-reducing bilateral salpingo-oophorectomy consistently reduces BC risk in *BRCA1* PV carriers but has a less marked effect in *BRCA2* PV carriers^30,31^, strongly supports the view that aberrant high progesterone levels in *BRCA1* PV carriers play a central role in the development of TNBC.

In addition to the proliferative effects of progesterone on the cells of origin for TNBC (i.e. basal and luminal progenitor cells)^24,25^, progesterone may exert its pro-carcinogenic potential via the modulation of immune surveillance given that immune tolerance mechanisms are shared by both cancer and pregnancy. The progesterone induced expression of B7-H4 (VTCN1), an onco-fetal immune tolerance checkpoint, contributes to progesterone-induced BC progression via CD8 + T cell exhaustion^32^. Progesterone may also be involved in the development and progression of metastatic high-grade serous ovarian cancer^33–35^, at least in part via progesterone-mediated suppression of natural killer (NK) cell cytotoxicity at the Fallopian Tube fimbriae, which are exposed to ultra-high concentrations of progesterone due to their close proximity of the ovaries^19^.

## Current modalities for assessing and monitoring TNBC risk

Primary cancer prevention implies that in the organ at risk (i.e. breast) there are neither macroscopic nor microscopic signs of cancer or pre-cancer. Significant amounts of research focus on the detection of the earliest signs of cancer, utilising circulating cells^36,37^ or circulating cell-products (e.g. cancer-specific altered DNA^9,38–40^, RNA^41^ or proteins^42,43^). However, none of these efforts have so far convincingly demonstrated a clinical benefit over existing imaging-based strategies, such as mammography or breast MRI. These imaging-based methods often lead to overdiagnosis^44,45^ and do not effectively detect TNBC and other BCs with poor prognosis, which have higher mortality rates compared to screen detectable cancers^46,47^.

PRS, which can be used to individualise BC risk prior to clinical manifestation, also have substantial limitations. A commonly used PRS, consisting of 313 common genetic variants preferentially predicts risk for estrogen-receptor positive BCs^48^. A new PRS, based on analysis of a more diverse cohort led by the US National Cancer Institute (Confluence), may help to overcome this issue^49^. PRS is being used in conjunction with other risk factors, such as age, to measure changing risk over time. Nevertheless, due to the nature of single nucleotide polymorphisms, a PRS per se cannot be used to monitor individual risk in response to an intervention.

Two parameters that directly assess breast tissue and may reflect cancer risk have gained some attention: (i) mammographic density is associated with BC risk, however, the association is weaker and has not been evaluated extensively in women 35 years of age or younger^50^. Mammographic density also depends on menstrual cycle^51^. Most importantly, women with TNBC tend to have lower mammographic density, less MRI-assessed fibroglandular tissue, and lower background parenchymal enhancement compared to non-TNBC^14^. Therefore, although suitable for monitoring risk modulation in older women on SERMs^52,53^, imaging-based risk monitoring in young *BRCA1* PV carriers at risk for TNBC is unlikely to be safe and feasible. (ii) Ki67, a marker for cell proliferation, has been used to assess SERM efficacy^54,55^. However, Ki67 expression in normal breast tissue is weakly associated with BC risk^56^. Additionally, it provides only a snapshot of current proliferation in breast tissue, is dependent on the menstrual cycle, and is difficult to assess in small breast biopsies with limited numbers of epithelial cells, leading to limited interlaboratory reproducibility^57^.

## Progesterone Receptor Modulators for prevention

Progesterone Receptor Modulators (PRMs; also called antiprogestins), such as mifepristone, play a pivotal role in reproductive health through their effects on the cervix and uterus^58^. PRMs have also demonstrated effects on breast epithelial cell proliferation^20,59–61^ and show BC preventive effects in mice^61,62^.

PRMs are currently in use for emergency-^63^ or on-demand contraception and are well-established for use in medical abortion^64–66^. Their efficacy for treating symptoms related to uterine fibroids^67^ and other reproductive health conditions^68^ was demonstrated many years ago. When administered at appropriate dosages and suitable delivery methods, PRMs like mifepristone offer a woman-centred contraceptive option with additional health benefits, supporting autonomy, while offering added non-contraceptive health benefits. Based on existing data, long-term treatment with mifepristone has found to be safe^69,70^ and is undergoing further exploration^71^.

One particular aspect of safety relates to the endometrium. PRMs have previously been observed to impact the endometrium but effects differ across PRM type, dose, timing of administration within the menstrual cycle, duration, and possibly also ethnicity^72^. Most of the evidence has been generated for mifepristone with less evidence for the other PRMs such as ulipristal acetate (UPA; now discontinued as a treatment for fibroids) and others that were under clinical development. Follicular phase administration has little effect on the endometrium, but depending on the dose used ovulation may be delayed or inhibited. Continuous administration of PRMs in low doses that do not affect ovulation can induce endometrial effects that are potentially contraceptive^73^. Prolonged use of doses that inhibit ovulation has led to concerns about endometrial hyperplasia due to unopposed estrogen. However, the endometrial changes that occur are a distinct histological entity known as PR modulator-associated endometrial changes (PAEC)^74^. Specifically, PAEC reveals an antiproliferative state and the endometrial glandular epithelium shows reduced mitotic activity compared to the proliferative phase although extensive cystic glandular dilatation is seen. Evidence indicates that PAEC regresses upon cessation of treatment^75^. Recent evidence demonstrates that three months of mifepristone exposure does not induce molecular changes associated with endometrial carcinogenesis^76^.

A summary of current indications and potential side effects of mifepristone are displayed in Table 1.

**Table 1:** Side effects of mifepristone in studies for a range of indications

Indication	Treatment dose, duration	Potential adverse effects	Endometrium	Liver function	Refs
Contraception	2–200 mg daily/weekly/ monthly, up to 12 months	Nausea, breast distention, pelvic/abdominal pain, bloating and headache, have been reported (at doses of 10–50 mg weekly).	PAEC (5 mg daily use for 6 months)^126^.	Isolated reports of elevated transaminases^127,128^, returned to normal within two months.	^126–135^
Emergency contraception	Single dose 10–50 mg	Nausea, vomiting.		N/A	^58,136^
Uterine fibroiids	2.5–50 mg daily or every other day, for 3–12 months	Headache, nausea, vomiting, mood swings, diarrhea, decreased libido, weakness, fatigue, hot flashes, nervousness.	Endometrial hyperplasia, PAEC (dilated cystic glandular formations). No atypia or malignancy.	Isolated reports of elevated transaminases.	^137^
Endometriosis	5–100 mg daily for 3–6 months	Nausea, vomiting, fatigue, hot flashes.	PAEC.	Isolated reports of elevated transaminases.	^137^
Obstetrics: Cervical ripening at term, induction of labor, delivery of still birth	Single dose 50–200 mg (recommended dose)	Hypoglycemia in newborn.		N/A	^138,139^
Induced abortion, management of missed miscarriage, cervical pretreatment before surgical abortion	Single dose 200–600 mg (recommended dose)	Urticarial skin rash, self-resolving.		N/A	^140–143^
Meningioma	200 mg daily for prolonged use (recommended dose)	Fatigue, hot flashes, breast tenderness, nausea, mood swings, rash, partial alopecia, edema and biochemical hypothyroidism.	Endometrial hyperplasia (no atypia).	Not affected (evaluated in few studies).	^144^
Cushing syndrome	300–1200 mg daily for prolonged use (recommended dose)	Hyper/hypokalemia, hyper/hypotension, hyponatremia, low cortisol levels, mineralocorticoid deficiency, maculopapular rash (in patients with hypercortisolism), edema, hyper/hypoglycemia, nausea/vomiting, headache, fatigue, secondary hypothyroidism.	Endometrial hyperplasia (no atypia).	Three reported cases of drug-induced liver injury (cholestatic liver injury) in patients with Cushing^145^.	^137,144^

*PAEC* PR modulator-associated endometrial changes; *N/A* not available

Accumulating evidence suggests that PRMs can prevent breast cancers, including poor prognostic TNBCs.

Although UPA is also highly effective^60,77^, its association with liver injuries^78^ precludes its use for BC risk reduction.

A downstream effector of progesterone – RANKL – can be targeted with antibodies such as denosumab (Amgen), which is in clinical use to treat bone loss^79^. Unlike mifepristone^68^, denosumab is not known to be lipophilic and, hence, would not be enriched in the fat-containing breast tissue. Targeting RANKL has been shown to prevent mammary gland cancer in mice^22,80^ but has no effect on contralateral breast cancer risk in postmenopausal BC patients^81^. The BRCA-P study is currently evaluating the BC risk reducing effect of denosumab in women carrying a germline *BRCA1* pathogenic variant (EudraCT 2017-002505-35 and NCT04711109). The acceptance rate is, however, considerably impaired by potential participants’ wishes to become pregnant and the fear of side effects.

Compared with denosumab, mifepristone has several features that may make it considerably more acceptable to young *BRCA1* PV carriers in the context of a prevention trial. First, mifepristone is taken orally at home and does not require clinic-based injections, conferring a meaningful practical advantage over denosumab (given as a subcutaneous injection every six months) that is likely to improve both recruitment and long-term adherence. Second, because low-dose mifepristone has well-documented contraceptive efficacy, it could simultaneously serve as the required contraception during the trial period - a prerequisite in any prevention trial involving reproductive-age women - thereby reducing the perceived burden of participation compared to alternative prevention drugs, which necessitates separate contraception. Third, unlike denosumab, mifepristone is anticipated to reduce the risk of both breast cancer and ovarian cancer in *BRCA1* PV carriers, given accumulating evidence that elevated or heightened progesterone responsiveness contributes to ovarian cancer risk in this population^33–35^. The prospect of a single oral agent that may simultaneously lower risk across both organs is a compelling patient-relevant argument that denosumab cannot offer. Fourth, short-term hormonal modulation with mifepristone has recently been shown not to induce oncogenic endometrial changes in *BRCA1/2* PV carriers^76^, providing direct reassurance about safety in this specific population.

## The role of the epigenome in assessing and monitoring TNBC risk

### The role of the epigenome in cancer development

Historically, cancer was considered a purely genetic disease, with the presence of somatic structural DNA alterations considered nearly conclusive evidence for the presence of a cancer^82^. It is now clear that DNA mutations including pathogenic driver mutations are present in normal tissues and are particularly evident in hormone-sensitive tissues like the endometrium^83,84^ and breast^85^, even at a very young age. Cancers can develop in the absence of DNA mutations, purely triggered by epigenetic alterations^86^ and epigenetic modifiers like those suppressing the activity of the polycomb repressor complex 2 component EZH2 frequently drive cancer formation^87^. Indeed, DNA methylation (DNAme) at promoter regions occupied by the polycomb repressor complex 2, belonging to the so called polycomb group target genes (PCGTs), is one of the most prevalent findings across all cancer entities^88–90^. Together data indicating that PCGT gene expression is essential for cellular differentiation^91^ and that methylation of promoter CpG islands leads to an almost irreversible gene suppression^92^, suggest DNAme at PCGT gene promoters is an essential factor in cancer development as it locks stem/progenitor cells in an undifferentiated state^93^.

The most unifying model of cancer development available today is that both exogenous factors and factors triggered by genetic predispositions drive the division of epithelial (stem/progenitor) cells and thereby increase cancer risk. For BC, exogenous factors include progesterone-containing hormone therapy^26^, which is associated with development of BCs with poor prognoses. Importantly, known genetic risk factors indirectly influence exposure to factors driving proliferation in specific at-risk cells. *BRCA1* deletion in granulosa cells in mice^94–96^ and *BRCA1* pathogenic variants in humans alter hormone levels (notably elevating luteal phase progesterone)^17,18,20^, immune function^19^ and microbiome composition^97^. These exogenous and genetically-triggered factors collectively promote proliferation of certain cell types and may ultimately lead to cancer formation^21,98^. They are integrated at the level of the epigenome, where integration depends on individual genotypes (i.e. single nucleotide polymorphisms; SNPs) which impact on DNAme patterns (i.e. methylation quantitative trait loci; meQTLs) in a cell-type-specific manner^99^. Most importantly, the epigenome is dynamic and malleable over time^20,93,100^.

### Monitoring cancer field defects

Tumors arise from a field of molecularly altered epithelial cells of a specific type – a “field defect” comprising DNAme and somatic DNA mutation – that creates a permissive environment for malignant evolution. Field defects mostly occur without visible, morphological changes. Quantifying and monitoring cancer risk could be achieved through identification of these field defects.

Field defects for cervical, ovarian, breast and lung cancers have been identified. Cervical cancer field cancerization has been studied most extensively due to accessible at-risk tissue prior to disease onset, allowing prediction of (pre)cancer development years in advance through DNAme- tests^101–103^. Most recently, DNAme markers assessed in samples with normal cytology were shown to predict 80% of invasive cervical cancers within six years following sample collection^102^.

Breast field cancerization has also been described: expression of the retinoic acid receptor beta (RAR-*β*) was absent in normal adjacent breast tissue^104^ due to DNAme of the *RARB* gene^105,106^. A large component of field cancerization is a consequence of stem/progenitor cell proliferation triggered by certain conditions^17,20,21^ which drive the accumulation of DNAme at PCGTs^88,91^. A DNAme-based mitotic clock, which reflects replicative age by focusing on promoter CpG sites that localize to PCGTs and are unmethylated in 11 different foetal tissue types, is universally accelerated in cancer, including pre-cancerous lesions (both of the breast and lung), and is also accelerated in normal breast tissue adjacent to cancers^20,107,108^. Importantly, this process appears to be reversible up to a certain stage, by removing the primary proliferative stimulus (e.g. with PRMs)^20^.

**Fig. 2:**
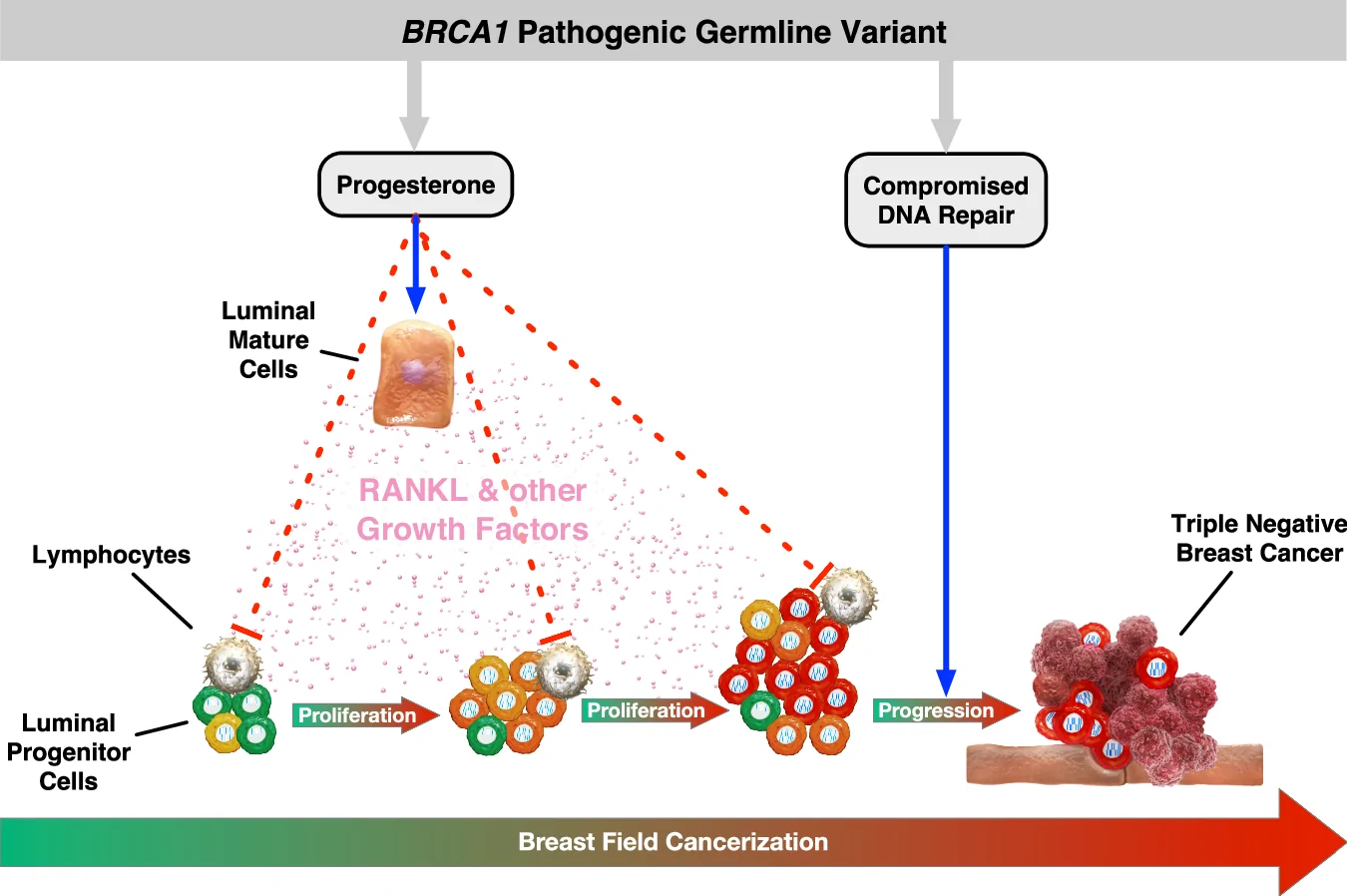
Illustration of field cancerization. Progesterone triggers (i) the release of RANKL (Receptor Activator of Nuclear Factor Kappa-B Ligand) in hormone receptor (HR)-positive mature luminal cells leading to increased proliferation and thus accelerated mitotic ageing in HR-negative luminal progenitor cells (this process is marked by increasing DNA methylation at polycomb repressor complex 2 target sites; indicated by blue lines in the luminal cell nuclei); and (ii) a reduction in immune-surveillance^32^. Both effects result in increased cancer risk. This risk is further enhanced by other cell-autonomous (compromised DNA repair) and non-cell-autonomous factors^20^. The risk state for individual cells increases from green to yellow to orange to red.

An example of field cancerization is illustrated in Fig. 2^20^: high progesterone levels from combined hormone therapy^26^ or a *BRCA1* germline PV^17,18,20^ trigger progesterone-receptor positive luminal mature cells to release RANKL and other factors that, in a paracrine fashion, activate the proliferation of estrogen- and progesterone-receptor negative luminal progenitor cells eventually leading to formation of TNBC^21,22,25,80,109^. Mechanisms to reduce the luminal progenitor cell population are not yet fully established, but treatment with PRMs may exert a direct effect^20^ and an indirect effect by enhancing NK cell cytotoxicity^19^.

### Cancer risk signatures in epithelial cells

As discussed above, DNAme signatures in normal epithelial at-risk tissue are indicative of cancer field defects and are associated with the development of cervical^102,103^, breast^20,110–112^, ovarian^113,114^ and endometrial^115^ cancers. Breast cancer field defects can be assessed in fine needle aspirates (FNA). These field defects can also be identified in easy-to-access epithelial surrogate tissues which have similar features to the epithelial cells from which the actual cancer is derived. These features could include cell-type (i.e. epithelial), embryological origin and hormone sensitivity. Epithelial cells from the cervix or buccal mucosa (inner cheek) are therefore relevant surrogate tissues in the case of breast cancer.

Utilizing DNAme signatures, there is evidence that the field defects can be identified in cervicovaginal and buccal samples, making them suitable for assessing risk for breast^116,117^, ovarian^118^, endometrial^119^ and lung cancers^120^. For example, the WID-BC, a BC risk test based on DNAme markers measured in cervicovaginal samples^116^ had an AUC of 0.81 (95% CI: 0.76–0.86) in an independent external validation dataset with 113 cases and 225 controls. In comparison, the PRS_313_^121^, analysed in 314 cases and 816 controls, had an AUC of 0.67 (95% CI: 0.64–0.71). Preliminary evidence suggests that the WID-BC can predict BC development up to 5 years before diagnosis^116^.

### Monitoring cancer risk with malleable markers

Small proof of principle studies have demonstrated that the disease-associated DNAme signatures can change dynamically due to specific interventions such as exposure to PRMs^20^ or smoking cessation^120^. Data from a window trial shows that mifepristone reduces both (i) DNAme markers reflective of stem/progenitor cells’ replicative age as well as (ii) the proportion of luminal progenitor and basal cells in normal breast tissue of healthy women without a *BRCA1/2* PV and in a proportion of *BRCA1/2* PV carriers^20^. These DNAme markers are higher in normal breast tissue adjacent to TNBC compared to normal breast tissue from age-matched cancer-free women and increase in almost all TNBCs when compared to normal adjacent tissue indicating that these markers are good intermediate surrogate markers. Whether risk indicating DNAme signatures measured in an epithelial surrogate sample, such as the WID-BC test, reflect the efficacy of mifepristone in reducing cancer risk over time has not yet been studied.

Serial breast biopsies are not standard of care and are not a practical solution for biomarker monitoring in a prevention trial. Circulating tumour-derived nucleic acids - whether cell-free DNA (cfDNA) or microRNAs - depend critically on shedding from (pre-)malignant cells and are inherently insensitive in a true primary prevention context where no tumour has yet formed. Fine needle aspiration (FNA) of the breast, whilst less invasive than a core biopsy, requires a clinic visit and trained personnel and is not yet standard of care in asymptomatic *BRCA1* PV carriers. Accessible epithelial surrogate tissues - cervicovaginal and buccal cells - share cell type, embryological origin, and hormone sensitivity with breast epithelium and can be obtained through simple non-invasive self-sampling at home. The WID-BC test, a DNAme signature measured in cervicovaginal samples, achieved an AUC of 0.81 in independent external validation and can predict breast cancer development up to five years before diagnosis^116^. Proof-of-principle from a mouse model demonstrates that mifepristone-induced DNAme changes in the cervix parallel the reduction in mammary tumour incidence^62^, directly validating the surrogate tissue approach as a pharmacodynamic readout. We therefore propose the following hierarchy of biomarker approaches for a mifepristone prevention trial in *BRCA1* PV carriers: (i) cervicovaginal and/or buccal DNAme signatures as the primary non-invasive endpoint; (ii) breast FNA-derived DNAme in a subset of willing participants to validate surrogate tissue signals against tissue-of-origin; and (iii) circulating liquid biopsy approaches (cfDNA methylation, miRNA panels) as exploratory endpoints, acknowledging their current sensitivity limitations in a prevention setting.

Recent findings from a mouse model using medroxyprogesterone acetate and 7,12-dimethylbenzanthracene to induce mammary tumours provide compelling evidence that epigenetic signatures can reflect cancer risk and serve as surrogate markers for monitoring preventive interventions. Treatment with mifepristone reduced mammary cancer incidence by more than 50%, a reduction that was paralleled by decreased epigenetic alterations both in the tissue at risk (normal mammary gland, indicating field cancerization) and in distant, non-target tissues such as the cervix, indicative of field defect^62^. These results strongly support the continued investigation of epigenetic biomarkers in accessible tissues to monitor the efficacy of cancer prevention strategies – particular of mifepristone - in humans.

## The vision: personalised TNBC prevention in *BRCA1* PV carriers

**Fig. 3:**
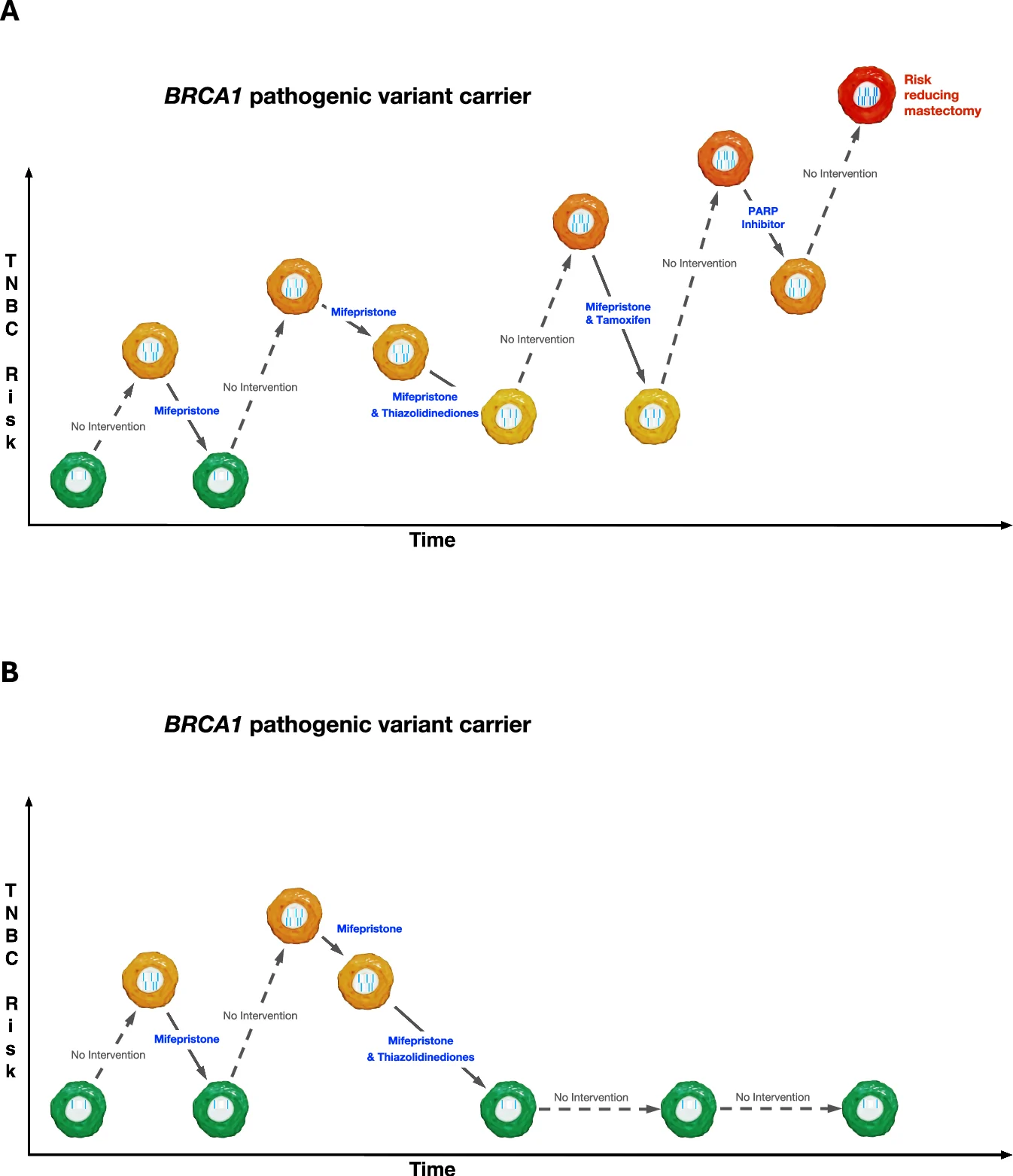
Approach to TNBC prevention in *BRCA1* PV carriers using the PRM mifepristone and potential complementary approaches alongside real-time risk assessment. The blue bars in the centre of the cells reflect DNA methylation as a reflection of replicative age (the higher the number of blue bars, i.e. DNA methylation at PCGTs, the higher the replicative age of these cells). The risk state for individual cells increases from green to yellow to orange to red. **A** Scenario illustrating gradual development of mifepristone resistance where additional preventive treatments could temporarily offset resistance and sustain preventive efficacy; **B** Scenario showing sustained and high mifepristone sensitivity, where primary prevention remains effective without the need for complementary therapies.

Our vision for personalised TNBC prevention includes *predicting* TNBC-risk in real time through the analysis of DNAme signatures in breast tissue (obtained through biopsy or more fine-needle aspiration which is more acceptable) or, eventually, in a more easily accessible epithelial surrogate sample. These markers would enable the ongoing assessment of primary prevention efficacy, for example, with mifepristone. They would also allow for *personalised* adjustments, such as changes in dosage, frequency or even medication choice which in addition to mifepristone includes drugs which have shown potential for breast cancer prevention (e.g. selective estrogen receptor modulators or thiazolidinediones^122^ or which are specifically active in *BRCA1* PV carriers (e.g. PARP inhibitors)^123^. Engagement with the patient community and integration of the aspects that matter most to them into the final solution, around risk communication and ease/invasiveness of sample collection and drug administration, would enhance the *participatory* nature of these endeavours (Fig. 3).

## Trial design and challenges

**Fig. 4:**
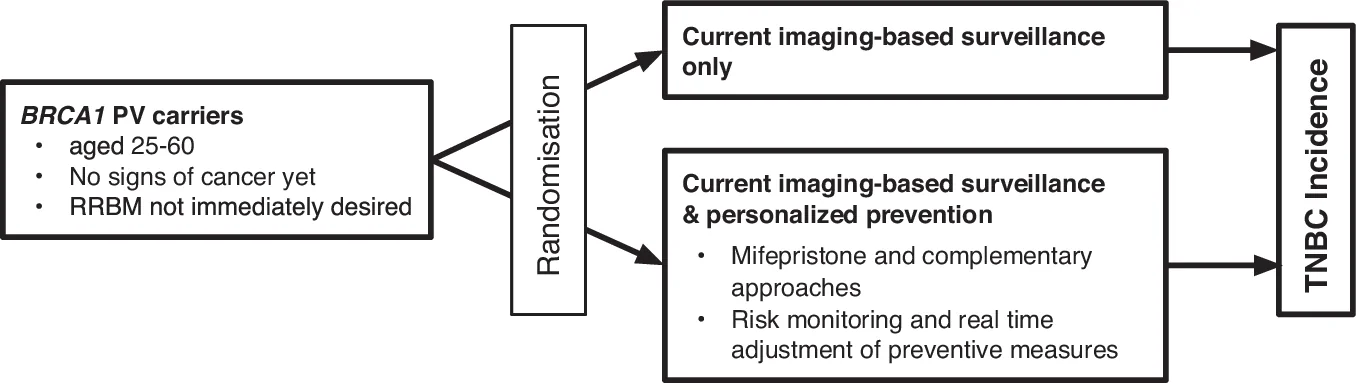
Proposed trials design. Proposed design for a trial assessing a personalised primary prevention approach utilising the progesterone receptor modulator mifepristone.

Figure 4 shows the proposed design for a definitive study aimed at testing a personalised primary TNBC prevention approach in *BRCA1* PV carriers.

Detailed discussions around the clinical trial design, including randomisation, control groups, statistics, inclusion/exclusion criteria, contraception, early BC detection, and risk communication during the trial, and more, are needed. In addition, a critical question is which intermediate surrogate markers reflective of the real time risk should be implemented and, most importantly, how these should be validated before inclusion in a clinical trial. The general criteria for such markers are as follows:

(i) They should demonstrate a gradual increase with increasing risk across a range of age and disease statuses, as measured in normal breast tissue, normal breast tissue adjacent to cancer, and cancer tissue. We note that the lack of longitudinal, population-based collection of normal breast tissue limits the scope of specific assessment and validation possible.
(ii) They must clearly indicate either a positive or negative response to an intervention.

Consensus from the scientific and regulatory communities on the extent of evidence supporting a biomarker’s effectiveness would be needed before embarking on a prevention study.

In light of the accrual challenges experienced by the BRCA-P trial and the current state of evidence for mifepristone in breast cancer prevention, a randomised phase IIB proof-of-concept trial with a short-term biomarker endpoint is a more immediately feasible first step than a large-scale randomised controlled trial (RCT) powered for TNBC incidence. Such a phase IIB trial would serve to assess drug acceptability, safety, and preliminary pharmacodynamic response, and could inform the design of a subsequent definitive prevention trial. The duration of drug intervention in such a trial would ideally be biomarker-guided - adapted in response to the individual’s risk trajectory as measured by intermediate surrogate markers - rather than fixed at a predetermined timepoint.

An important consideration in selecting the biomarker endpoint for such a phase IIB trial is its biological relevance to the specific cancer subtype at risk. Background parenchymal enhancement (BPE) on breast MRI has been proposed as a potential short-term endpoint given that annual breast MRI is standard of care in *BRCA1* PV carriers. However, a recently published large pooled analysis of the Breast Cancer Association Consortium (BCAC) demonstrated that mammographic density (percentage density) shows a markedly attenuated - effectively null - association with breast cancer risk specifically in *BRCA1* PV carriers (OR per SD: 1.02, 95% CI 0.78–1.34), in contrast to *BRCA2* carriers (OR per SD: 1.55, 95% CI 1.08–2.24), and attributes this finding to the predominance of the triple-negative subtype in *BRCA1*-associated cancers^124^. Because BPE and mammographic density are inter-related imaging surrogates of fibroglandular tissue, and because women with TNBC characteristically have lower BPE and mammographic density compared to women with non-TNBC, BPE is likely to be insensitive - or even directionally misleading - as a pharmacodynamic endpoint for monitoring TNBC risk reduction in *BRCA1* PV carriers. For this reason, DNA methylation (DNAme)-based biomarkers in accessible surrogate tissues (see below) are preferred as intermediate endpoints in this population.

### Challenges of mifepristone

Due to fact that mifepristone has been shown to have an acceptable safety profile without liver toxicity, it is the most suitable PRM for consideration in the context a future breast cancer prevention trial.

Despite evidence from mouse models and a very limited number of window trials, the evaluation of mifepristone^20,59^ for breast cancer prevention has not been extensive. Data generated with mifepristone are supported with trials using other PRMs such as ulipristal acetate^20,60^. Large-scale evidence from adjuvant trials similar to that which exists for SERMs – agents that are central components of adjuvant breast cancer treatment – are unlikely to become available for PRMs. Data from ongoing contraceptive trials with mifepristone add to its long-term safety profiles^71^.

Evaluation of mifepristone’s effectiveness could start within the context of a randomised clinical trial amongst very high-risk women. To walk towards this already challenging starting point, additional hurdles have to be overcome. Chiefly, these revolve around the negative political and cultural perceptions of mifepristone, which is primarily known as an abortion drug, and the limited access to it in countries where abortion laws are restrictive. Additionally, funding and regulatory challenges exist. The interest from the pharmaceutical industry is low, in part because of the association of mifepristone with abortion, but also because mifepristone is off-patent and prevention trials can be multi-national, long and expensive. Who will fund such a trial in the absence of pharma interest? Consensus from regulators on the design of clinical trials, and the biomarkers used within them, would also be needed to ensure results can be used to support implementation.

Nevertheless, when compared with denosumab (the agent being tested in the BRCA-P trial), mifepristone has several important potential advantages for acceptability and feasibility. These are elaborated in the context of a possible stepwise trial approach described in the previous section.

An additional topic requiring clarification is which drugs, when applied at low doses for short periods, could affect mifepristone-resistant cells (see Fig. 3 panel A). Several options exist and could potentially be applied in the same trial. These include intermittent fasting, a ketogenic diet/supplement (unpublished data), PARP inhibitors, which have particularly high antineoplastic activity in *BRCA1* PV carriers compared to non-carriers^125^, and – still very speculatively – thiazolidinediones, which may help prevent breast cancer by activating PPARγ, which reduces cell proliferation, promotes cellular differentiation, and improves insulin and inflammatory signalling pathways^122^. The effectiveness of these approaches, particularly if applied in combination, will need to be considered.

New, personalised approaches to primary breast cancer prevention are urgently needed and could be developed by building on the last 15 years of research into the pathogenesis of TNBC in *BRCA1* PV carriers and methods to monitor risk. However, a community effort is needed to reach this goal of a new personalised cancer prevention approach. A consensus workshop, held in Innsbruck in September 2024, began this community effort. This group – the International Mifepristone Breast Cancer Prevention (IMBCP) Group – will now broaden its reach and continue to work towards ensuring that the necessary discussions take place, ultimately bringing about tangible change for women at the highest risk.Table 2

**Table 2:** The role of progesterone receptor modulators in breast cancer prevention

Study	Preventive effects	Type of PRM	Type of study	Methodology	Main Findings	Ref
Barrett et al., 2025	Yes	Mifepristone	Mouse model	Medroxyprogesterone acetate and 7,12-dimethylbenzanthracene was used to induce mammary gland cancers in mice	Mifepristone reduced mammary gland cancers by >50%	^62^
Lee et al., 2023	Likely	Onapristone and ulipristal acetate	Tamoxifen-insensitive mouse model	Genetically modified C3(1)-TAg mice, which develop aggressive ER+ mammary tumors, were used to evaluate Z-endoxifen (5 mg/kg/day) as monotherapy and combined with selective progesterone receptor modulators ulipristal acetate (16.7 mg/kg/day) or onapristone (20 mg/kg/day). (*n* = 10–12 mice per group).	Ulipristal acetate (81 mg/day) significantly delayed tumor onset (*p* = 0.03; latency 114 days, *p* = 0.005), but did not reduce tumor growth or multiplicity; Onapristone (98 mg/day) showed no preventive effect.	^146^
Ranjan et al., 2021	Yes	Mifepristone and telapristone	Mouse	Ovariectomized mice were randomized into four groups (9 mice/group): sham, estrogen (0.3 mg) and progesterone (30 mg), estrogen and progesterone with telapristone (30 mg), and estrogen and progesterone with mifepristone (30 mg). After 14 days of treatment, mammary glands were collected for RNA sequencing and splicing analysis.	Estrogen and progesterone significantly increased MSC and luminal mature fractions and reduced luminal progenitor cells in breast. Moreover, they strongly activate Wnt signaling, most prominently in MSC. Progesterone receptor antagonists reversed progesterone driven tumorigenic effects in mammary cells.	^147^
Lee et al., 2021	Yes	Telapristone acetate, ulipristal acetate, and mifepristone	BRCA1 knockout mouse model	Mice were treated with (30 mg/90 days) of mifepristone (*n* = 13), telapristone acetate (n = 10), or ulipristal acetate (*n* = 6) via subcutaneous pellets. The control group had 28 mice, Tumorigenesis was monitored, and histological analysis was performed at euthanasia. RNA was extracted from benign mammary glands for sequencing and gene expression analysis.	Ulipristal acetate significantly reduced tumor development (*p* = 0.04), achieving a 37% reduction in adenocarcinoma compared with an 18% reduction by Telapristone and no effect by mifepristone. Ulipristal acetate delayed tumor onset by >2 months	^148^
Lee et al., 2016	Yes	Telapristone acetate	Rat/mouse	Mice (*n* = 10–11) were treated with subcutaneous medroxyprogesterone acetate(MPA) (25 mg/pellet) alone or with telapristone acetate (25 mg/pellet), followed by weekly gavage of DMBA (1 mg) for 4 weeks. In the rat model (*n* = 10–11), a single injection of MNU (50 mg/kg) was followed by implantation of medroxyprogesterone acetate (MPA), progesterone, or telapristone acetate (identical doses to the mice study). Tumor formation and histology were assessed at study termination (65 days).	In mouse, Telapristone acetate opposed the pro-hyperplastic effects of MPA (*p* = 0.002) In a rat mammary carcinogenesis model induced by MNU, MPA and progesterone increased tumor incidence and tumor burden compared with controls. Telapristone acetate significantly reduced tumor incidence when combined with MPA (*p* = 0.037) or progesterone (*p* = 0.032). The lowest tumor incidence (36%) was observed in rats treated with progesterone plus Telapristone acetate. Tumor burden was significantly reduced by Telapristone acetate in combination with progesterone compared with progesterone alone (*p* = 0.042).	^149^
Communal et al., 2016	Yes	Ulipristal acetate	Mouse xenograft model	Breast tissue from 6 control and 5 *BRCA1* PV carriers was xenografted into 4-week-old ovariectomized mice. Treatments included control, estradiol (1.5 mg/pellet), estradiol + progesterone (25 mg/pellet), or estradiol + progesterone + ulipristal acetate (30 mg/pellet) (*n* = 4 mice/group). Mice were sacrificed after 28 days for analysis.	Ulipristal acetate inhibited progesterone driven proliferation in *BRCA1* PV breast tissue.	^150^
Communal et al., 2012	Unclear	Ulipristal acetate	In vitro (Human) and xenograft athymic mouse model	Normal human breast epithelial were xenografted in athymic mice (*n* = 4) treated with 20 mg UPA per pellet for 28 days.	Ulipristal acetate had no effect on the mitotic index of xenografted human breast tissue exposed to gonadal hormones at levels similar to those in normal women. In vitro findings indicated that Ulipristal acetate reduced the proliferation of normal breast cells but promoted growth in breast cancer cells.	^151^
Wiehle et al., 2011	Yes	Telapristone acetate	Rat	Rats at the age of 50 days were injected with N-methyl-N-nitrosourea to initiate mammary carcinogenesis, and 6 days later, Telapristone acetate (CDB-4124) pellets (30.0 and 3.0 mg/pellet) were implanted subcutaneously for a duration of 3 months. (*n* = 16 to 20 rats/group)	Telapristone acetate (CDB-4124) demonstrated strong chemopreventive efficacy in rat mammary cancer models, with the following outcomes: - Increased tumor latency from (66 ± 24 to 87 ± 20 days) (*P* < 0.02) - Reduced tumor incidence from 85% → 35% (*P* < 0.001) - Decreased tumor multiplicity by from 3.0 → 1.1 tumors/animal (*P* < 0.001) - Lowered tumor burden from (2.6 → 0.26 g/animal) - Prevented spontaneous premalignant lesions at 200 mg/kg over 24 months	^152^
Gonzalez-Suarez et al., 2010	Yes	Mifepristone	Mouse	Mice were treated with 10 mg/kg muRANK-muFc, PBS, or zoledronic acid. Tumor growth was monitored by palpation, and mice were euthanized when tumors exceeded a certain size. Proliferation assays were conducted using BrdU, and progesterone and mifepristone (10 mg/kg) were administered to examine hormonal effects. (*n* = 24-32).	Mifepristone reduced 98% of progesterone-induced proliferation (*P* < 0.001 versus control) whereas RANK-Fc reduced proliferation by 82% (*P* < 0.001 versus control).	^22^
Kariagina et al., 2010	Yes	Mifepristone	Organoid	T47D-Y, T47D-YA, and T47D-YB cells were cultured in serum-free media, treated with R5020 (20 nM) ± Iressa (500 nM) or mifepristone (100 nM) for 24 hours, followed by 25 nM Areg for 30 minutes.	Estrogen and progesterone act synergistically to drive mammary epithelial proliferation, with combined estrogen and progesterone treatment inducing robust proliferation in ducts (24%) and lobules (39%), compared with minimal effects of estrogen alone (3-4%) and no effect of progesterone alone. In primary mammary organoids, Mifepristone significantly reduced the proliferative effect of Areg + progesterone, indicating that active progesterone receptor signalling is required for hormone-induced epithelial growth Mifepristone reduced the proportion of S-phase cells back to basal levels, confirming suppression of cell-cycle progression.	^153^
Lim et al., 2009	No	Mifepristone and onapristone	In vitro	Mammary epithelial subsets from human tissue, including *BRCA1* PV carriers, were analyzed using flow cytometry. Cells were cultured in Matrigel with supplemented medium, including mifepristone or onapristone.	The clonogenic activity of luminal progenitor cells from *BRCA1* PV carriers was not inhibited by mifepristone or onapristone despite higher progesterone receptor expression in these cells.	^154^
Poole et al., 2006	Yes	Mifepristone	Brca1/p53-deficient mouse model	Mice were implanted with a pellet containing 35 mg/60-day constant-release mifepristone (*n* = 14) or a placebo pellet (*n* = 4). The study assessed the impact of mifepristone on tumor development and the role of *BRCA1* in regulating progesterone receptors.	Mifepristone prevented mammary tumorigenesis in Brca1/p53-deficient mice, with no palpable tumors detected up to 12 months of age in treated animals (*n* = 14), compared with 100% tumor penetrance and a median tumor latency of 6.6 months in control mice (*n* = 25). Mifepristone significantly reduced ductal branching and alveologenesis	^61^
Li et al., 1995	Yes	PRM ZK114043	Mouse	Whole organ cultures of mammary glands from estradiol/progesterone-primed virgin mice maintained in a serum-free medium with aldosterone, prolactin, insulin, and hydrocortisone. The cultures were treated with the PRM ZK114043 (5 ng/ml) for 4 days.	ZK114043 promoted differentiation in mammary cells. ZK114043 showed potent antiproliferative and pro-differentiation effects in the normal mammary gland. ZK114043 strongly inhibited epithelial DNA synthesis ZK114043 induced a differentiated secretory phenotype, with increased lipid accumulation in alveolar cells.	^155^

Study	Preventive effects	Type of PRM	Study Duration	Type of study	Sample Size (*N*) / Main Inclusion and Exclusion criteria	Methodology	Main Findings	Ref
Simões et al, 2025	Yes	Ulipristal acetate: 5 mg daily	12 weeks	Window-of-opportunity clinical trial	*N* = 24 Inclusion: Premenopausal women (25–45 years old) with regular cycles and *BRCA1/2* mutation or women at moderate to high familial BC risk. Exclusion: history of breast, uterine, cervical or ovarian cancer; pregnancy/breastfeeding, prior breast enhancement, or use of hormonal therapies, steroids, anticoagulants, or CYP3A4 inhibitors; liver disease/cirrhosis	Effect of ulipristal acetate on surrogate markers of breast cancer risk in 24 premenopausal women	Significant reduction in Ki67 (8.2% → 2.9%, P < 0.0001) alongside a drop in progesterone levels (36 → <3 nmol/L, P < 0.0001), effectively suppressing the luteal phase. Decreased epithelial area in lobules and reduced acinar structure size following treatment. Luminal progenitor cells significantly reduced (from 43% to 30%, P < 0.001), with no changes in luminal mature or basal cells 50 genes differentially expressed (log2FC > 1.5, P < 0.05) after the treatment.	^156^
Utjés et al., 2024	Yes	Mifepristone: 50 mg every other day	8 weeks	Secondary Outcomes of a RCT	*N* = 16 Inclusion: Healthy premenopausal women (18–43 years old) with regular cycles, no treatment contraindications. Exclusion: history of cancer, pregnancy/breastfeeding	Breast biopsies were collected from 16 women at baseline and after two months of treatment with 50 mg mifepristone every other day, yielding 32 paired samples. Breast cancer-related signatures and progesterone receptor signalling pathways were analyzed.	27 differentially expressed genes identified, mainly linked to extracellular matrix remodelling. Mifepristone-induced gene signature in vivo closely matched in vitro findings Gene signature associated with breast carcinogenesis and progesterone receptor status	^157^
Bartlett et al., 2022	Yes	Mifepristone: 50 mg every other day; UPA: 5 mg daily	12 weeks	RCT	*N* = 44 Inclusion: Healthy premenopausal women (18–45 years old) with regular cycles and confirmed BRCA1/2 mutation (or non-carrier controls). Exclusion: recent hormonal medication, pregnancy/breastfeeding, history of cancer, or prior bilateral salpingo-oophorectomy	Two cohorts were studied: the first received mifepristone (50 mg every other day), and the second received ulipristal acetate (5 mg daily). Effects on breast cancer risk markers, including cell composition, proliferation, *TP53* mutation, and methylation, were assessed in women with and without *BRCA* PV.	Progesterone levels are higher in luteal phase in *BRCA1* PV carriers, suggesting progesterone as a target for cancer risk reduction. Triple Negative Breast Cancers show elevated DNA methylation signatures reflecting higher mitotic age and higher proportion of luminal progenitor cells supporting the idea that luminal progenitor cells with high replicative age represent a predisposition to malignant transformation. Mifepristone reduces both mitotic age and luminal progenitor cell proportion in normal breast tissue in all controls and 64% of *BRCA1/2* PV carriers. Moreover, Ulipristal acetate produces similar effects, validating the role of progesterone pathway targeting. Mifepristone decreases TP53 mutation frequency and the number of mutations in normal breast tissue of mitotic-age responders.	^20^
Westhoff et al., 2022	Yes	Ulipristal acetate: 10 mg daily	12 weeks (84 days)	RCT	*N* = 25 Inclusion: Healthy women (18–39 years old, BMI ≤ 35 kg/m²) with regular cycles, no baseline breast mass/ovarian cysts, and no contraindications to COCs. Exclusion: recent exogenous hormones, pregnancy, breastfeeding, or interacting medications.	Women aged 18–39 received ulipristal acetate (10 mg daily) or combination oral contraceptives for 84 days, with breast cell proliferation and background parenchymal enhancement monitored.	Ulipristal acetate reduced Ki67 by 84% (p = 0.040), while the COC group showed an 8% increase (p = 0.85) BPE scores decreased from 3 to 1 (p = 0.008) with ulipristal, with no change in the COC group	^60^
Engman et al., 2008	Yes	Mifepristone: 50 mg every other day	12 weeks	RCT	*N* = 28 Inclusion: Premenopausal women referred for leiomyoma surgery (due to menorrhagia/pressure symptoms). Exclusion: recent steroidal hormone therapy, history of cancer, abnormal baseline breast/pelvic imaging, urgent surgery needs, postmenopausal FSH levels, or significant systemic/hormonal dysfunction.	30 women, scheduled for surgical treatment of leiomyomas, randomized to either 50 mg mifepristone or placebo every other day, for 3 months.	Mifepristone significantly reduced Ki-67 index after 3 months (P = 0.012) with lower inter-individual variability. Mifepristone did not affect cortisol but increased serum testosterone levels. Mifepristone reduced breast soreness and swelling but increased the incidence of hot flushes.	^59^

*RCT* Randomized Controlled trial; ER, Estrogen Receptor; *EP* Estrogen and Progesterone (EP); *MSC* Mammary stem cell; *LP* Luminal Progenitor; *PV* Pathogenic Variant; *TAg* SV40 Large T Antigen; *DMBA* Dimethylbenzanthracene; *MPA* Medroxyprogesterone Acetate; *MNU* N-Methyl-N-nitrosourea; *muRANK* murine RANK (Receptor Activator of NF-κB); *muFc* murine Fc region; *PBS* Phosphate-Buffered Saline; *RANK-FC* recombinant fusion protein consisting of the extracellular ligand-binding domain of RANK coupled to an immunoglobulin Fc region, which acts as a soluble decoy receptor to neutralize RANKL and thereby inhibit RANK signaling; *PRM* Progesterone Receptor Modulator; *BPE* Background Parenchymal Enhancement.

## Data Availability

No datasets were generated or analysed during the current study.
